# Dual Role of Insulin-Like Growth Factor (IGF)-I in American Tegumentary Leishmaniasis

**DOI:** 10.1155/2021/6657785

**Published:** 2021-03-29

**Authors:** Carolina de O Mendes-Aguiar, Camilla Lopes-Siqueira, Fabrício Pettito-Assis, Márcia Pereira-Oliveira, Manoel Paes de Oliveira-Neto, Claude Pirmez, Alda Maria Da-Cruz, Hiro Goto

**Affiliations:** ^1^Laboratório Interdisciplinar de Pesquisas Médicas, Instituto Oswaldo Cruz, FIOCRUZ, Rio de Janeiro, Brazil; ^2^Instituto de Medicina Tropical do Rio Grande do Norte, Universidade Federal do Rio Grande do Norte, RN, Brazil; ^3^Instituto de Medicina Tropical de São Paulo, Faculdade de Medicina, Universidade de São Paulo, São Paulo, Brazil; ^4^Instituto Nacional de Infectologia Evandro Chagas, FIOCRUZ, Rio de Janeiro, Brazil; ^5^Disciplina de Parasitologia, DMIP, Faculdade de Ciências Médicas, Universidade do Estado do Rio de Janeiro, Brazil

## Abstract

**Background:**

Cytokines and growth factors involved in the tissue inflammatory process influence the outcome of *Leishmania* infection. Insulin-like growth factor (IGF-I) constitutively present in the skin may participate in the inflammatory process and parasite-host interaction. Previous work has shown that preincubation of *Leishmania* (*Leishmania*) *amazonensis* with recombinant IGF-I induces accelerated lesion development. However, in human cutaneous leishmaniasis (CL) pathogenesis, it is more relevant to the persistent inflammatory process than progressive parasite proliferation. In this context, we aimed to investigate whether IGF-I was present in the CL lesions and if this factor may influence the lesions' development acting on parasite growth and/or on the inflammatory/healing process. *Methodology*. Fifty-one CL patients' skin lesion samples from endemic area of *L.* (*Viannia*) *braziliensis* infection were submitted to histopathological analysis and searched for *Leishmania* and IGF-I expression by immunohistochemistry.

**Results:**

In human CL lesions, IGF-I was observed preferentially in the late lesion (more than 90 days), and the percentage of positive area for IGF-I was positively correlated with duration of illness (*r* = 0.42, *P* < 0.05). IGF-I was highly expressed in the inflammatory infiltrate of CL lesions from patients evolving with good response to therapy (2.8% ± 2.1%; median = 2.1%; *n* = 18) than poor responders (1.3% ± 1.1%; median: 1.05%; *n* = 6; *P* < 0.05).

**Conclusions:**

It is the first time that IGF-I was detected in lesions of infectious cutaneous disease, specifically in American tegumentary leishmaniasis. IGF-I was related to chronicity and good response to treatment. We may relate this finding to the efficient anti-inflammatory response and the known action of IGF-I in wound repair. The present data highlight the importance of searching nonspecific factors besides adaptive immune elements in the study of leishmaniasis' pathogenesis.

## 1. Introduction

Leishmaniases are diseases caused by protozoan parasites of the genus *Leishmania*, endemic in around 88 countries. In Brazil, *L.* (*Viannia*) *braziliensis* is the main species for American tegumentary leishmaniasis (ATL), causing injuries ranging from benign cutaneous to disfiguring mucosal lesions [1]. After promastigote inoculation in the skin, these parasites interact primarily with innate host elements and growth factors present in this site. Once established the infection, the inflammatory infiltrate in the cutaneous leishmaniasis (CL) is characterized as chronic inflammation, with granuloma with or without necrosis, and the presence of macrophages, plasma cells, and lymphocytes [2]. The disease's progression is not directly correlated to progressive parasite growth in the lesion site, with few parasites being detected mainly in chronic cases [3]. Instead, cell-mediated immunity has essential participation in CL pathogenesis. Chronic CL lesions are composed of an increased number of activated CD69^+^ T [4] and regulatory CD4^+^CD25^+^FOXP3^+^ IL-10-producing T cells [5, 6], granzyme A CD8^+^ cytotoxic T cells, or even proinflammatory CD4^+^ IFN-*γ*-producing T cells [6]. Further, in a recent transcriptomic study in skin samples of ATL patients, delayed or no cure was correlated to the higher expression of gene sets related to the cytolytic pathway [7]. These findings exemplify the complexity of CL immunopathogenesis.

In the skin, different innate elements and growth factors participate at the inoculation site of the parasite. Insulin-like growth factor (IGF)-I is one of them, and we have been studying its participation in *Leishmania*-vertebrate host interaction. It is a hormone that acts as an autocrine and/or paracrine element, being essential to maintain body homeostasis. It can be detected in the serum, but it has a widespread distribution in tissues [8]. Many cells, including macrophages, produce IGF-I [8–10]. Different IGF-I serum levels were associated with the pathogenesis of melanomas [11], HPV infection [12], and psoriasis [13]. In cutaneous tissues, IGF-I has a central role in wound healing [14]. In leishmaniasis, previous studies have demonstrated the effect of IGF-I in inducing *in vitro* proliferation of different species of *Leishmania* [15, 16]. *In vivo*, in mouse model of CL, an increase in lesion size and the number of viable parasites after infection with IGF-I preactivated promastigotes of *L. amazonensis* was observed [17]. IGF-I induces arginase activation, which in turn activates *Leishmania* promastigotes [18]. Moreover, infection with IGF-I-preactivated *Leishmania* leads to an increase in lesion size in mice, due to the expansion of the inflammatory infiltrate and parasite growth, suggesting that IGF-I may contribute to cell migration besides parasite proliferation [17]. However, in ATL patients with mucosal and disseminated forms, IGF-I serum levels were lower than in simple CL and healthy controls [19].

IGF-I can potentially interact with *Leishmania* parasites in the initial phase of infection. Nevertheless, it is still unknown whether IGF-I has any role in the later stages of infection and whether this growth factor may contribute to leishmaniases' pathogenesis. Therefore, we aimed to investigate whether IGF-I was present in the CL lesions and if this factor may influence the development of the lesion acting on parasite growth and/or on the inflammatory/healing process.

## 2. Subjects and Methods

### 2.1. Growth Curve of *Leishmania* spp. in the Presence of IGF-I

*L.* (*V.*) *braziliensis* (MHOM/BR/1975/M2903) promastigotes were maintained at 25°C, in Schneider's insect medium (Gibco, Thermo Fisher Scientific Inc, MA, USA) supplemented with 10% heat-inactivated fetal calf serum (Gibco, USA) and 200 IU of penicillin per mL, and 200 *μ*g of streptomycin (Sigma Chemical Company, St. Louis, Mo, USA) per mL, and grown until stationary phase before using in *Leishmania* growth curve analysis. The stationary phase promastigotes were distributed in triplicate into 24-well plates (5 × 10^5^ parasites/well) in a final volume of 1.0 mL of Schneider's insect medium (Gibco, USA) supplemented with 2% FCS and antibiotics, with or without 50 ng/mL recombinant human IGF-I (rIGF-I, R&D Systems, USA) and maintained during the experimental period.

### 2.2. Patients

The patient groups were composed of 37 men and 14 women, all of them living in endemic area of *L. braziliensis* transmission. The median age was 35 [18-57] years. The patients were classified according to the duration of illness at the moment of diagnosis as early group when the appearance of the lesions was less than 30 days before; intermediate, in the 30-60-day interval; late, more than 90 days. The patients were also grouped by having a good response (complete epithelization three months after the end of therapy) or poor response (no complete healing of lesions three months after the end of treatment or development of secondary lesions).

The following criteria were used for leishmaniases diagnosis: (i) type of lesion and epidemiological data compatible with ATL; (ii) positive delayed-type hypersensitivity reaction to leishmanial antigens; (iii) detection of serum anti-*Leishmania* antibodies; and (iv) detection of *Leishmania* parasites in lesion by microscopic examination of histological sections or by culture in NNN-modified medium. Patients were treated with pentavalent antimony (*N*-methyl-glucamine, Glucantime). All procedures were approved by the Ethical Committee of the Fundação Oswaldo Cruz, Ministério da Saúde, Rio de Janeiro, Brazil, and informed consent was obtained from each subject.

The skin fragment was obtained for diagnosis before treatment, and it was taken from the border of the cutaneous lesion. The fragment was divided into three parts: one was fixed for histopathology analysis, the second one was cryopreserved for immunohistochemistry analysis, and the last one was used for *Leishmania* DNA detection by PCR. Fragments of skin lesions were not taken sequentially after treatment for ethical reasons.

### 2.3. Immunohistochemistry

Skin fragments were frozen in OCT resin (Tissue Tek; Sakura Finetek, Torrance, CA, U.S.A.), were cut into 3-4 *μ*m thick sections and mounted on microscope slides (silanized slides; DakoCytomation, Carpinteria, CA, USA). To detect IGF-I expression, the slides were fixed in acetone : methanol : formalin (19 : 19 : 2) and rehydrated in Tris-Saline Buffer (TBS) pH 7.6. The procedure was performed according to the Envision Double Staining kit (Dako, USA) manual. Briefly, endogenous enzymes were blocked with Endogenous Enzyme Block regent, and then, the slides were incubated with the first primary polyclonal rabbit anti-human IGF-I antibody (1 : 200; GroPep Limited–Adelaide, Australia) followed by incubation with the mouse and rabbit antibody conjugated to Polymer/HRP reagent. This first antibody was developed by DAB+ Chromogen. For the second part of immunostaining, beginning with the blocking step, the Doublestain Block reagent was used. The anti-*Leishmania* mouse serum was used as a second antibody and was added diluted 1 : 8000. This anti-*Leishmania* serum was obtained at two months of infection from BALB/c mice infected in the footpad with 10^6^ stationary phase *L. amazonensis* promastigotes [20]. Then, mouse and rabbit antibodies conjugated to Polymer/AP reagent were used, and permanent Red Chromogen developed the second reaction. Between the steps, the slides were washed with TBS pH 7.6, and the antibodies were diluted in BSA 2%-Tris-HCL 0.01 M pH 7.6 and incubated for 30 minutes, at room temperature each. At the end of the procedure, the slides were counterstained with Meyer's hematoxylin (Merck, Germany). For control purposes, the specific antibodies were omitted. The slides were examined under a light microscope (Nikon, Nikon, Eclipse E600, Japan).

#### 2.3.1. Immunohistochemistry Analysis

Two independent observers analyzed the slides. All fragment area was analyzed, and the IGF-I and anti-*Leishmania* staining were classified in only IGF-I detection, IGF-I and *Leishmania* antigen coexpression, or only *Leishmania* antigen detection. Twenty-five cases in which IGF-I was present were photographed. Five representative areas were photographed using Cool Snap-Pro Color (Media Cybernetics Inc, USA), and the photos were analyzed by Image-Pro Plus® Software (Media Cybernetics Inc, USA). The percentage of the positive area was measured for IGF-I and *Leishmania* sp. antigen staining.

### 2.4. Statistical Analysis

Mann-Whitney test, Kruskal-Wallis test, and Spearman correlation were utilized (GraphPad Software; San Diego, CA, USA) using the software GraphPad Prism version 6. Results were considered statistically different when the *P* value was ≤ 0.05.

## 3. Results

### 3.1. *Leishmania* (*Viannia*) *braziliensis in vitro* Growth upon IGF-I Stimulus

**Figure 1:**
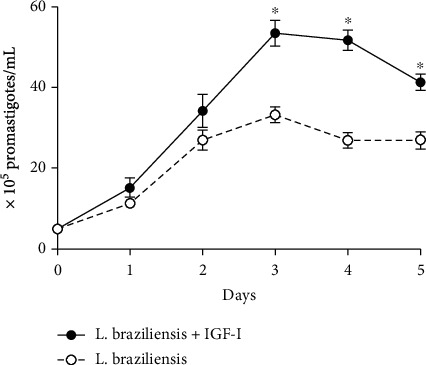
Effect of insulin-like growth factor (IGF)-I on *Leishmania* (*Viannia*) *braziliensis* promastigote proliferation. In vitro *L. braziliensis* growth curve in the presence (black circle) or absence (white circle) of 50 ng/mL rIGF-I. Live promastigotes were counted every day for five days. The points represent the mean of the three experiments, and the bars represent standard deviation. ^∗^*P* < 0.05.

In a previous study, the effect of IGF-I on parasite growth *in vitro L. braziliensis*-infected human monocytic cell line THP1 was inconclusive [19]; thus, we first investigated whether IGF-I can influence the *L. braziliensis* growth *in vitro*. The addition of rIGF-I in promastigote cultures in physiological concentration increased the *L. braziliensis* proliferation compared with controls without IGF-I (Figure 1).

### 3.2. IGF-I Was Present in Cutaneous Leishmaniases Lesions

**Figure 2:**
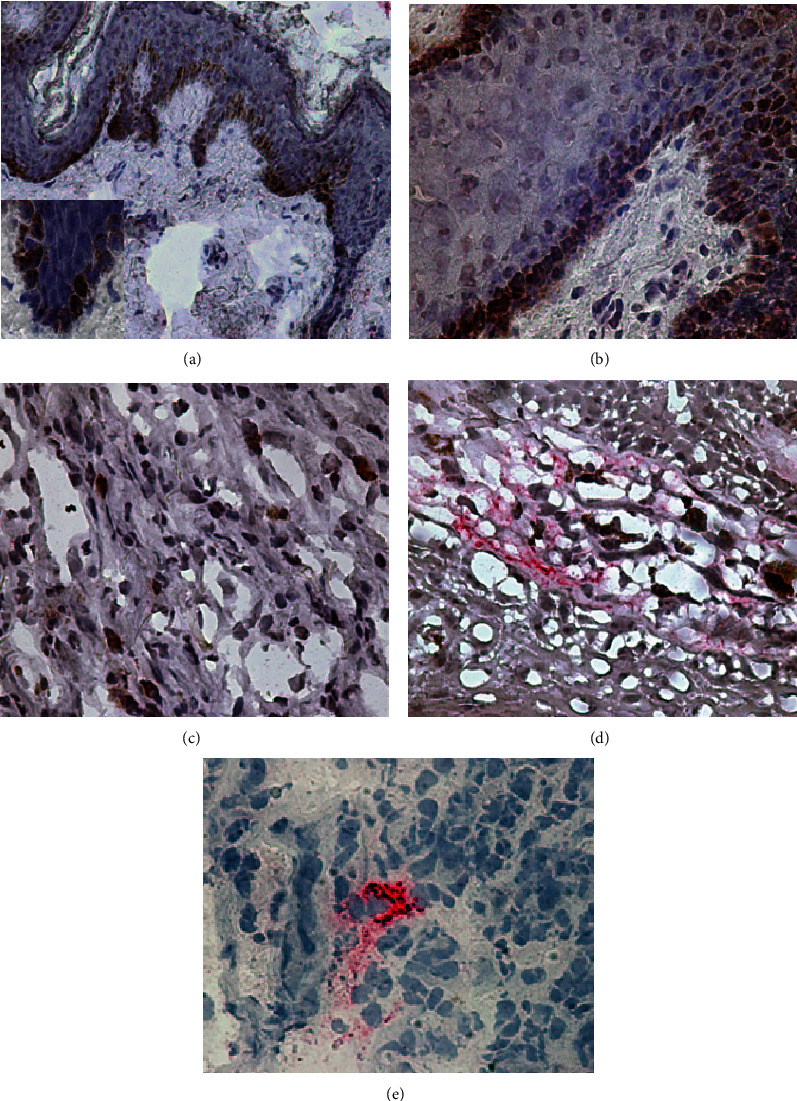
*In situ* detection of insulin-like growth factor (IGF)-I and *Leishmania* antigens in the inflammatory infiltrate of cutaneous leishmaniases lesions caused by *L.* (*Viannia*) *braziliensis.* IGF-I (brown) and *Leishmania* antigens (red) are expressed in normal skin (a) and cutaneous leishmaniases lesions (b–e). IGF-I is present in the basal layer in normal skin (a) and in CL lesions (b). In CL lesions, IGF-I is present in the dermis (c). IGF-I was observed close to *Leishmania* antigens in the dermis (d). *Leishmania* antigens were observed in the dermis (e). Representative photos of immunohistochemical analysis. Original magnification ×400.

Next, we investigated the presence of IGF-I in fifty-one CL lesions by immunohistochemistry. IGF-I was present in 70.5% of cases (*n* = 36). Histologically, IGF-I was spread in the dermis and epidermal basal lamina (Figures 2(b) and 2(c)). In normal skin (control; *n* = 3), IGF-I was seen only in the basal lamina (Figure 2(a)).

Concomitantly with IGF-I immunostaining, we carried out the *Leishmania* antigen detection. *Leishmania* antigens were detected in 84.3% of cases (*n* = 43). Isolated *Leishmania* antigen detection was found in 29.5% of cases (*n* = 15). *Leishmania* antigens were observed in the extracellular matrix and cell foci in the dermis (Figure 2(d)). *Leishmania* antigen detection decreased with chronicity: 92.8% of patients (13/14) in the early group; in 89.4% of patients (17/19) in the intermediate group; and in 72.2% of patients (13/18) in the late lesion group (Table 1). IGF-I and *Leishmania* antigens were observed in the dermis of the same lesion area in 28 cases. However, IGF-I and *Leishmania* antigens' colocalization was not observed.

### 3.3. The Duration of Illness and Treatment Response in relation to the Area of Expression of IGF-I

To evaluate the relationship of the presence of IGF-I with clinical parameters, 36 IGF-I positive cases were grouped according to the duration of illness or treatment response.

IGF-I seems to have a relationship to the chronicity of the lesions since the number of cases expressing IGF-I increases with the disease's progression. The IGF-I was detected in 64.2% of patients (9/14) in the early lesion group, in 68.4% of patients (13/19) in the intermediate group, and in 77.7% of patients (14/18) in the late lesion group (Table 1). Moreover, percentage of positive area of IGF-I was also correlated with duration of illness (early lesion group: mean = 1.52% ± 1.12, median = 1.47%, *n* = 7; intermediate group: mean = 2.32% ± 2.47, median = 1.5, *n* = 10; late lesion group: mean = 3.18% ± 1.9, median = 3.12, *n* = 8; *r* = 0.42, *P* = 0.023) (Table 2).

To assess the relationship of IGF-I expression with treatment response (*n* = 24), we grouped patients in those having a good response (complete epithelization three months after the end of therapy) or having a poor response (no complete healing of lesions three months after the end of treatment or development of secondary lesions). IGF-I was detected in 75% of patients with good therapeutic response. Those with good response showed higher percentage of positive areas for IGF-I (good responder: mean = 2.8% ± 2.1, median = 2.1%, *n* = 18) when compared with poor response (poor responder: mean: 1.3% ± 1.1, median = 1.05%, *n* = 6; *P* = 0.03) (Table 2).

## 4. Discussion

In this work, we initially showed the enhanced proliferation of *L. braziliensis* promastigotes in the presence of IGF-I. Further, IGF-I was detected in human CL lesions, and for the first time, it was shown in lesions of an infectious cutaneous disease. A limitation of this study was the unfeasibility to obtain sequential material from individual patients due to ethical issues, and further, we had patients who had a different time of development of the overt disease at the moment of diagnosis. However, we were able to analyze the histopathological alterations relating them to the duration of disease and also to response to treatment. Then, the percentage of patients presenting IGF-I detectable in the lesion was higher in chronic lesions. It was also higher among good responders for treatment.

IGF-I interacts with several *Leishmania* promastigote species inducing proliferation [15–17]. This effect did not occur with IGF-II polypeptide, which shares 60% of similarity with IGF-I [15]. Here, we showed *L. braziliensis* promastigote growth in culture in the presence of a physiological concentration of IGF-I as previously observed for other *Leishmania* species. Thus, we would suggest that IGF-I might act at the beginning of infection interacting with promastigotes at the moment of *Leishmania* inoculation in the host's skin. With other species, amastigotes were also shown to proliferate in the presence of physiological concentrations of IGF-I [16] but inconclusive with *L. braziliensis* [19]. IGF-I binds to a receptor expressed on the *Leishmania* parasite surface, causing phosphorylation of certain proteins and increase the replicative rate of both parasite forms (promastigote and amastigote) in culture [16].

In this work, we analyzed the presence of IGF-I in CL lesions. It is the first data on the presence and influence of *in situ* IGF-I in human cutaneous infectious disease. In CL lesions, IGF-I was found spread throughout the dermis, basal lamina, and epidermis differing from normal skin where it was seen only in basal lamina. IGF-I's capacity to continuously stimulate keratinocyte growth [9] may contribute to altering the tissue architecture and, at first glance, maybe connected to acanthosis often present in CL lesions [21]. In psoriasis, a cutaneous inflammatory disease, IGF-I contributes to lesion severity stimulating continuous keratinocyte growth [22]. Thus, we hypothesize that the presence of IGF-I in CL lesions may influence the disease outcome.

IGF-I presence in CL lesions with different distribution and a constitutive presence of IGF-I in normal skin leads us to ask whether this factor could act in different phases of *Leishmania* infection. To address this question, we have performed a double immunostain to IGF-I and *Leishmania* antigens by immunohistochemistry. *Leishmania* antigens were detected mainly in recent lesions confirming previous observations [23]. In the mouse model, the infection with IGF-I preactivated *L. amazonensis* promastigotes induced larger lesions than non-pre-activated parasites [17]. The aggravation of mice CL lesions was due to an increase in the number of parasites along with intense cell migration. However, in the present study, we could not observe colocalization of IGF-I and *Leishmania* antigens in CL lesions suggesting another role for IGF-I in human ATL other than on parasite proliferation. In *in vitro* study, it was shown intrinsic IGF-I surrounding *L. major* within infected macrophages [24], but it could be due to the differences among *Leishmania* species and in this particular experimental conditions, very far from the lesion pathogenic process in the skin.

Here, we also correlated IGF-I expression with lesion chronicity and treatment response. Our results showed that area of IGF-I expression was positively correlated with duration of illness (chronicity). Many immune factors can contribute to chronic CL lesions in humans [6], where the lesion development is related instead to hypersensitivity than to susceptibility to parasite growth [25, 26]. In this context, increasing concentration of IGF-I in chronic lesions may suggest IGF-I's known participation in anti-inflammatory response [27, 28], counteracting persistent, and intense inflammation.

Analyzing the IGF-I area concerning treatment response, we observed higher IGF-I expression areas in patients with good response to the treatment. We may relate this finding to the efficient anti-inflammatory response and the known action of IGF-I in wound repair in the reepithelialization [29]. Retarded wound healing in mice diabetic lesions was associated with a delay in IGF-I expression [30]. In humans, a lack of IGF-I expression in diabetes mellitus ulcer contributes to a retarded wound repair [31]. Besides, IGF-I treatment helped to restore the normal expression of both matrix metalloproteinase (MMP)-9 and tissue inhibitors of metalloproteinase (TIMP)-1 in diabetic rats with skin ulcers [32], indicating an important role of IGF-I in healing. Furthermore, in CL patients, MMP-2 was associated with a satisfactory response to antimonial therapy, in conjunction with moderate amounts of IFN-*γ*, IL-10, and TGF-*β* [33]. It is conceivable to argue that IGF-I present in CL lesions can act in this net of soluble factors comprising cytokines and hormones involved in the homeostatic process, thus influencing the healing process.

In the pathogenesis of lesion in cutaneous leishmaniasis, inflammatory cytokines [34] and cytotoxic mechanisms [7] are involved that are modulated during development to cure. In this context, the interplay between interferon-gamma (IFN-*γ*) and IGF-I may determine the outcome of the disease. IFN-*γ* decreases IGF-I expression through inhibition of transcription of IGF-I mRNA [35], and IFN-*γ* is a cytokine that is important for parasite control but implicated in the lesion pathogenesis. In patients with CL in Brazil, expression of IFN-*γ* tends to decrease during the time, mainly in subjects with good response to treatment [36]. Thus, we may speculate that it may result in higher expression of IGF-I in patients with chronic evolution and a good response to treatment.

Our results suggest that IGF-I can play a dual role in CL lesions. IGF-I was detected more in chronic ulcers where may act as an anti-inflammatory factor and in lesions of good responders to treatment where the contribution of this hormone on wound healing would prevail. IGF-I's dual role in CL lesions could be explained by the complex pathogenesis of CL lesions, whereas in the same lesion, we can find areas of an intense inflammatory response and wound repair [34]. It is also observed regions with synthesis besides degradation of extracellular matrix granuloma, compatible with transient or reversible histopathological features occurring in CL lesions [37]. The interplay between IGF-I and other immune-inflammatory elements present in CL lesions may influence the disease outcome.

In the study of leishmaniases' immunity and pathogenesis, most approaches focus on the adaptive immune response that is undoubtedly relevant. However, the present data highlight the importance of searching nonspecific factors such as growth factors besides adaptive immune elements in the study of leishmaniasis' pathogenesis.

## Figures and Tables

**Table 1:** Insulin-like growth factor (IGF)-I or *Leishmania* antigen detection in lesions of cutaneous leishmaniases patients according to the duration of illness.

	% of positive cases	Duration of illness	% detection (*n*/*n*_total_)
Leishmanial antigens	84.3% (*n* = 43)	Early	92.8% (13/14)
		Intermediate	89.4% (17/19)
		Late	72.2% (13/18)

IGF-I	70.5% (*n* = 36)	Early	64.2% (9/14)
		Intermediate	68.4% (13/19)
		Late	77.7% (14/18)

Duration of illness: early = lesions with less than 30 days; intermediate = lesions with 30-60 days; late = lesions with more than 90 days.

**Table 2:** Area of expression of insulin-like growth factor (IGF)-I in the lesions of cutaneous leishmaniasis patients according to the duration of illness and response to treatment.

	Groups	IGF-I-area %		*P*
		Median	Mean ± SD	
Duration of illness	Early lesion (*n* = 7)	1.47	1.52 ± 1.12	^∗∗^ *r* = 0.42 0.023
	Intermediate (*n* = 10)	1.50	2.32 ± 2.47	
	Late lesion (*n* = 8)	3.12	3.18 ± 1.90	

Treatment response	Good responder (*n* = 18)	2.10	2.80 ± 2.10	^#^0.03
	Poor response (*n* = 6)	1.05	1.30 ± 1.10	

^∗∗^Spearman correlation, ^#^Mann-Whitney test.

## Data Availability

The data used to support the findings of this study are included within the article.
